# DOCK1 regulates the malignant biological behavior of endometrial cancer through c-Raf/ERK pathway

**DOI:** 10.1186/s12885-024-12030-1

**Published:** 2024-03-04

**Authors:** Shangdan Xie, Yanshan Jin, Jiakun Wang, Jingwei Li, Mengjia Peng, Xueqiong Zhu

**Affiliations:** 1 Zhejiang Provincial Clinical Research Center for Obstetrics and Gynecology, Department of Obstetrics and Gynecology, The Second Affiliated Hospital of Wenzhou Medical University, 325027 Wenzhou, Zhejiang China; 2 Department of Obstetrics and Gynecology, Taizhou Women and Children’s Hospital of Wenzhou Medical University, 317599 Taizhou, Zhejiang China

**Keywords:** c-Raf, DOCK1, Endometrial cancer, ERK, Mechanism

## Abstract

**Background:**

The effect of DOCK1 gene on the biological behavior of endometrial carcinoma cells and its related pathway has not been reported.

**Methods:**

The immunohistochemical method and western blot were utilized to analyze DOCK1 protein expression in endometrial tissues and cells, respectively. CCK-8, BrdU, transwell and flow cytometry were performed to analyze the effect of DOCK1 expression changes on the viability, proliferation, invasion, migration and apoptosis of endometrial cancer cells, respectively. The effects of DOCK1 gene on Bcl-2, MMP9, Ezrin, E-cadherin and c-RAF/ERK1/2 signaling pathway were evaluated by western blot. The xenograft models were constructed to analyze the effect of DOCK1 in vivo.

**Results:**

DOCK1 expression was increased in endometrial cancer tissues and cells compared with those in normal adjacent tissues and cells. DOCK1 knockout could inhibit the malignant biological behavior of endometrial cancer cells, while DOCK1 overexpression played the opposite effect. The expression of E-cadherin was upregulated and those of MMP9, Ezrin, Bcl-2, p-c-RAF (S338) and p-ERK1/2 (T202/Y204) were downregulated after DOCK1 knockout, while DOCK1 overexpression played the opposite effect. Additionally, Raf inhibitor LY3009120 reversed the function of DOCK1 on malignant biological behavior. In vivo experiment results showed that the growth and weight of transplanted tumors in nude mice were inhibited after DOCK1 knockout. The changes of E-cadherin, MMP9, Ezrin and Bcl-2 expressions in the transplanted tumors were consistent with those in vitro.

**Conclusion:**

DOCK1 could enhance the malignant biological behavior of endometrial cancer cells, which might be through c-RAF/ERK1/2 signaling pathways in vitro and in vivo.

**Supplementary Information:**

The online version contains supplementary material available at 10.1186/s12885-024-12030-1.

## Introduction

Endometrial cancer is one of the most frequent malignancies occurring in women, with about 97, 370 deaths and 417, 000 new cases worldwide every year [1]. The etiology of endometrial carcinoma is not clear, but it may be related to estrogen level, age and genetic susceptibility [2]. Many studies demonstrated that abnormal expression of many genes and dysregulation of related signaling pathways might foster the carcinogenesis and progression of endometrial cancer [3, 4]. The treatment of endometrial cancer consists of surgery, endocrine therapy and chemotherapy [5], but the efficacy for patients with advanced or recurrent cancer is not ideal. With the increasing incidence and mortality of endometrial carcinoma, it is pivotal to explore the mechanisms of cancer biological behavior, which has far-reaching significance for exploring biomarkers and targeted drugs applied in diagnosis and therapy.

All the 11 members of dedicator of cytokinesis (DOCK) family are guanine nucleotide exchange factors, each of which possesses distinct functions lied on their expression patterns and substrate specificity [6]. DOCK1 (also known as DOCK180), the first identified member of the DOCK family, is located on chromosome 10q26.2 and takes part in various biological processes, including embryonic development, axonogenesis and angiogenesis [7, 8]. The Rac Rho small GTPase is activated to participate in cytoskeletal rearrangements through the exchange from bound GDP to free GTP by DOCK1 in complex with engulfment and cell motility protein 1 (ELMO1) [9, 10]. It is reported that mammary gland degradation in mice depleted of both DOCK1 and Rac1 resulted in many dead epithelial cells that could not be rapidly cleared by phagocytes, suggesting that DOCK1 played a critical role in mediating phagocytosis of epithelial phagocytes [11]. Notably, DOCK1 might contribute to cancer development. For instance, downregulation of DOCK1 protein inhibited epithelial-mesenchymal transition (EMT) of bladder cancer cells [12]. Epidermal growth factor receptor (EGFR) elevated the invasive ability of glioblastoma through DOCK1-MLK3 signaling axis [13]. However, few studies investigated the function and mechanism of DOCK1 in gynecological cancers [14], and no research has been reported in endometrial cancer so far. Hence, the present study explored the molecular mechanism of DOCK1 in regulating biological behavior of endometrial cancer and hopes to provide a new direction and theoretical basis for the early diagnosis and potential treatment of endometrial cancer.

## Materials and methods

### Tissue samples

Tissues of endometrial cancer and normal adjacent endometrium were collected from 15 patients with endometrial cancer in the Second Affiliated Hospital of Wenzhou Medical University and the participants subscribed preoperative informed consent. The sections were deparaffinized with xylene and gradient ethanol. The present study was approved by the ethics committee of the Second Affiliated Hospital of Wenzhou Medical University.

### Immunohistochemical assay

The slices were placed at an oven of 65℃ for 1 h to soften the paraffin, followed by deparaffinization in isopropanol twice and gradient alcohol (100%, 95%, 90%, 85%, 80%, 75%) sequentially. Then the slices were put into sodium citrate buffer, boiled with microwave for antigen repair and were added with hydrogen peroxide solution for endogenous peroxidase inactivation. After blocking the slices with 3% bovine serum albumin (BSA) for 40 min, the corresponding primary antibodies (1:200 rabbit anti-human antibodies: anti-Bcl2 antibody, anti-DOCK1 antibody, anti-E-cadherin antibody, anti-Ezrin antibody, anti-MMP9 antibody) were added at 4℃ overnight. The next day, incubated with horseradish peroxidase-conjugated secondary antibody, stained with diaminobezidin (DAB) and hematoxylin solution. The staining results analysis was described in our previous study [15].

### Cell culture

Human normal endometrial epithelial cell EEC was obtained from Shanghai Whelab Bioscience Limited, cultured with MEM medium. Human endometrial cancer cell HEC-1 A and endometrial cancer cell Ishikawa were purchased from the Cell Bank of the Chinese Academy of Sciences (Shanghai, China), and were cultured in McCoy’s 5 A and DMEM medium, respectively. Rac1 GTPase inhibitor NSC23766 (Selleck, USA) was added into HEC-1 A and Ishikawa cells to analyze the effect on the c-Raf/ERK signaling pathway.

### Western blot assay

The total proteins were collected by lysis buffer with protease inhibitors. The detection procedure of specific proteins levels by western blot was listed in our previous study [16]. The primary antibodies (1:1000) were shown as follows: rabbit anti-Bcl2 antibody (Boster, China), rabbit anti-DOCK1 antibody (CST, USA), rabbit anti-E-cadherin antibody (Proteintech, USA), rabbit anti-Ezrin antibody (Proteintech, USA), mouse anti-GAPDH antibody (CST, USA), rabbit anti-MMP9 antibody (CST, USA), rabbit anti-tubulin antibody (ABclonal, China), rabbit anti-phospho-C-Raf (S338) antibody (CST, USA), rabbit anti-C-Raf antibody (CST, USA), rabbit anti-phospho-ERK1/2 (Thr202/Tyr204) antibody (CST, USA), rabbit anti-ERK1/2 antibody (CST, USA). The chemiluminescence method was used to display the bands.

### Construction assay of DOCK1 knockout (KO) cell lines with CRISPR-Cas9 technique

DOCK1 knockout Ishikawa and HEC-1 A cell lines were constructed via CRISPR-Cas9 method. Cell transfection was utilizing 2 µg sgRNA1, 2 µg sgRNA2 and 8 µL Lipofectamine 2000 (Invitrogen, USA) for 48 h and cell screening was using 2 µg/mL puromycin for 72 h. Afterwards, the cells were diluted into 1000/mL and dispensed into 96-well plates with 100 mL/well. Ultimately, western blot was conducted to evaluate the efficacy of knockout. DOCK1-KO group was treated with pSpCas9(BB)-2 A-Puro (Px459) plasmids containing DOCK1-sgRNAs. The sgRNA sequences were listed: DOCK1-sgRNA1-F: CAC CGA ATA GCT TCT AAA CAA GTG G; DOCK1-sgRNA1-R: AAA CCC ACT TGT TTA GAA GCT ATT C; DOCK1-sgRNA2-F: CAC CGC TAA TCG TGC TAG TTA ATT C; DOCK1-sgRNA2-R: AAA CGA ATT AAC TAG CAC GAT TAG C.

### Construction assay of DOCK1 overexpression cell line with instantaneous transfection technique

DOCK1 overexpression Ishikawa was transfected with the vector containing human DOCK1 cDNA (pCDNA3.1-DOCK1‐HA, Youbio Biosciences), and the control cell was transfected with the pCDNA3.1 vector (Youbio Biosciences) for 48 h. Then, cells were treated with ampicillin for 3 d and the efficacy was verified by western blot. In addition, 20 µM Raf inhibitor LY3009120 was added in DOCK1-overexpressing Ishikawa cells for 48 h to carry out the following assays.

### Assessment of cell viability and proliferation

Cell Counting Kit-8 (CCK-8) assay was utilized to detect cell viability and the cells were seeded into 96-well plates with 2, 000/well for adherence. The viabilities of cells with incubation for 0 h, 24 h, 48 h, 72 and 96 h were analyzed by the absorbance of 450 nm wavelength with a Microplate Reader (Bio Tek Instruments, Winooski, VT, USA). BrdU was supplemented into the cell medium for proliferation detection. The main procedure was as previously described [16]. Ultimately, the positive cells were observed under a microscope.

### Assessment of cell migration and invasion

Ishikawa (2 × 104 cells for migration and 5 × 104 cells for invasion) or HEC-1 A (5 × 104 cells for migration and 1 × 105 for invasion) cells in 100 µL serum-free medium were added into upper chambers (8.0 μm pore size; Costar, USA) without or with matrigel basement membrane matrix (BD, Biosciences, USA). The lower chambers were treated with 700 µL complete culture medium for 24 h. The migrated cells or invaded cells were treated with 4% paraformaldehyde for 30 min, dyed with crystal violet (Beyotime, China) and found under a microscope with a 200 × magnification.

### Assessment of cell apoptosis

5 × 105 cells were harvested and stained with Annexin V-FITC/PI detection kit (BD, Biosciences). The detailed procedure was shown in previous study ^16^. Then the percentage of apoptotic cells was evaluated by flow cytometry assay based on manufacturer’s instructions.

### Animal study

4–6 w old female BALB/c nude mice (Beijing Weitonglihua Sciences Co. Inc., China) were randomly divided into 2 groups (control group and KO group, *n* = 6) and each group was subcutaneously injected with corresponding 5 × 10^6^ Ishikawa control cells or DOCK1-KO cells into the left flank. Tumor growth of each mouse was measured once a week and the volume was determined as follows: (length × width2)/2. The mice were euthanized by pentobarbitol Sodiumat day 28, and then the tumors were weighed and collected for making tissue slices. The animal study was approved by Institutional Animal Care and Use Committee of Wenzhou Medical University.

### Statistical analysis

Graph Pad Prism 8.0 was used to evaluate the experimental data. Results with a normally distributed distribution were presented as mean ± SD. The Student’s t test was utilized to evaluate the difference between two groups, and when the data were normally distributed, one-way analysis of variance (ANOVA) was performed to assess the difference between multiple groups (more than two groups). The homogeneity test of variance was conducted using the Levene test. Non-parametric rank sum test was used to compare statistical difference for non-normally distributed data. The significance was shown by the P value of 0.05.

## Results

### DOCK1 protein expression is increased in endometrial cancer tissues and cells

**Fig. 1:**
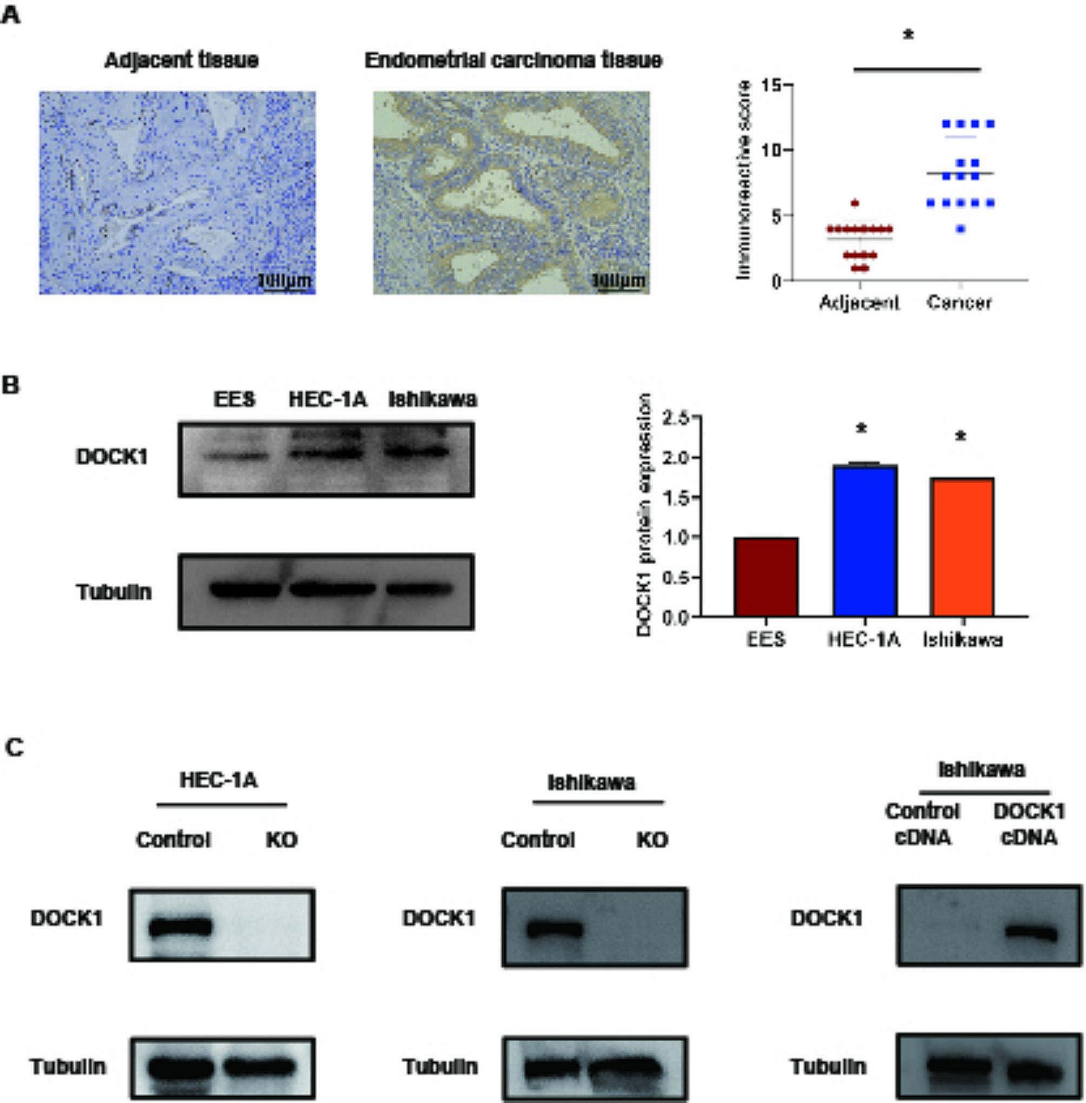
DOCK1 protein expression in endometrial carcinoma tissues and cells. (**A**) The protein expressions of DOCK1 in endometrial carcinoma tissues and adjacent tissues by immunohistochemical assay. (**B**) The protein expressions of DOCK1 in EES, HEC-1 A and Ishikawa cells by western blot. (**C**) The efficacy of DOCK1 knockout or overexpression on HEC-1 A and Ishikawa cells by western blot. *P < 0.05

Immunohistochemical technique was utilized to determine the DOCK1 protein expression in endometrial carcinoma tissues and adjacent normal endometrial tissues. As shown in Fig. 1A, DOCK1 expression was upregulated in endometrial cancer tissues in contrast to adjacent normal endometrial tissues (*P* < 0.05). Compared with normal endometrial cell EES, the DOCK1 expression was upregulated in HEC-1A and Ishikawa cells (*P* < 0.05, Fig. 1B).

HEC-1 A cell was chosen to knockout DOCK1 expression, and Ishikawa cell was selected to knockout and overexpress DOCK1 expression. The efficacy of DOCK1 knockout and overexpression was shown in Fig. 1C.

### DOCK1 enhanced the proliferation of endometrial cancer cells

**Fig. 2:**
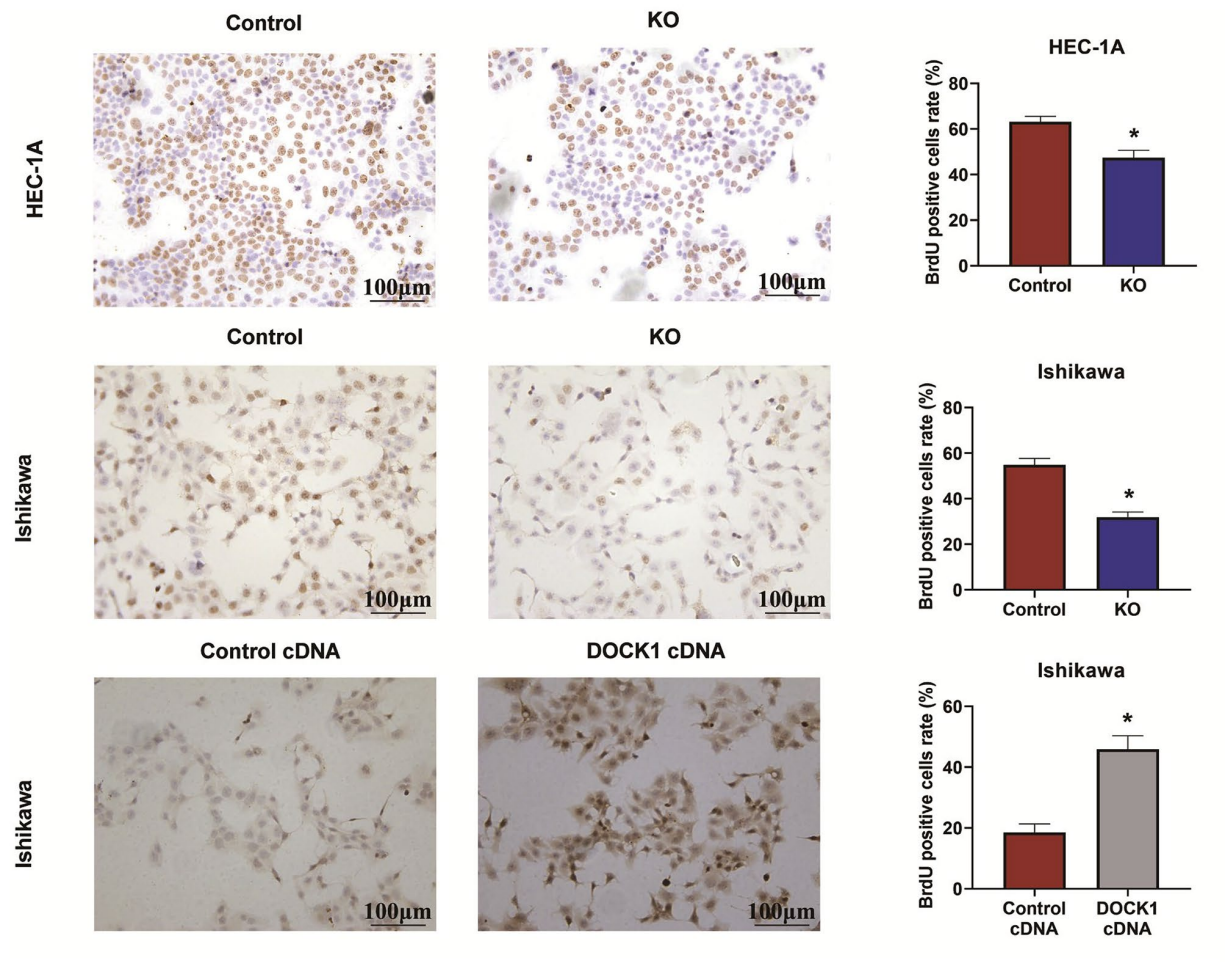
DOCK1 regulated the proliferation of endometrial cancer cells. *P < 0.05

As demonstrated in Fig. 2, the BrdU results displayed that the percentages of proliferative cells in DOCK1 KO groups were lower than those in corresponding control groups (*P* < 0.05), but DOCK1 overexpression promoted the proliferation of Ishikawa cells (*P* < 0.05).

### DOCK1 promoted the migration and invasion of endometrial cancer cells

**Fig. 3:**
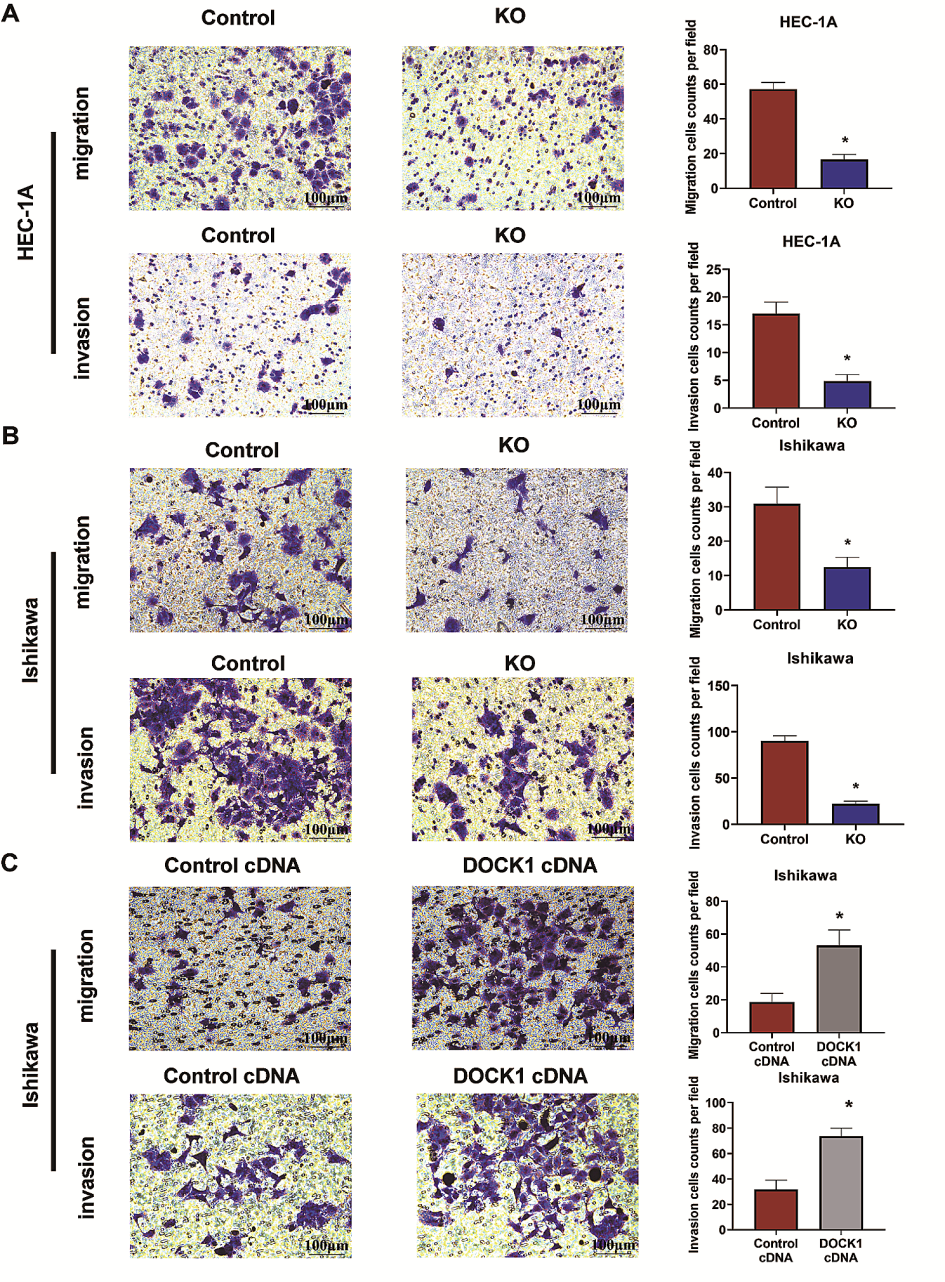
DOCK1 mediated the migration and invasion of endometrial cancer cells. (**A-B**) The effect of DOCK1 knockout on the migration and invasion of HEC-1 A and Ishikawa cells. (**C**) The effect of DOCK1 overexpression on the migration and invasion of Ishikawa cells. *P < 0.05

Transwell method was conducted to detect the mobility of HEC-1 A and Ishikawa cells. The results demonstrated that the inhibition of DOCK1 could weaken the cell migrative and invasive abilities (*P* < 0.05), while the overexpression of DOCK1 elevated the migration and invasion of Ishikawa cells (*P* < 0.05, Fig. 3).

### DOCK1 regulated the apoptosis of endometrial cancer cells

**Fig. 4:**
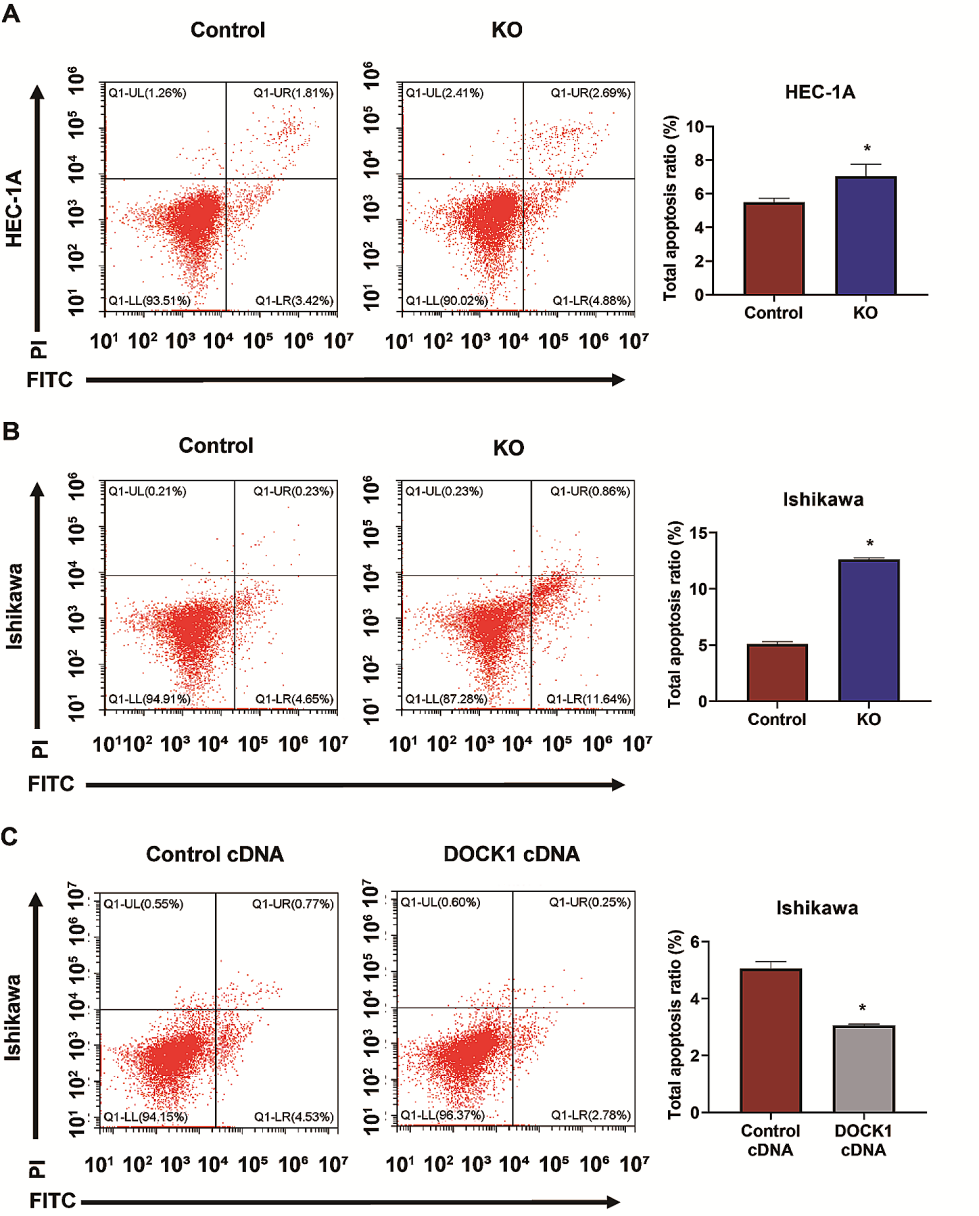
DOCK1 modulated the apoptosis of endometrial cancer cells. (**A-B**) The effect of DOCK1 knockout on the apoptosis of HEC-1 A and Ishikawa cells. (**C**) The effect of DOCK1 overexpression on the apoptosis of Ishikawa cells. *P < 0.05

Annexin V-PI/FITC staining assay was carried out to determine the number of apoptotic cells. As shown in Fig. 4A-B, DOCK1 knockout elevated the apoptosis of HEC-1 A and Ishikawa cells (*P* < 0.05). Conversely, the apoptotic rate of Ishikawa cells with DOCK1 overexpression was decreased (*P* < 0.05, Fig. 4C).

### DOCK1 modulated the cell malignant biological behavior via c-Raf/ERK signaling pathway

**Fig. 5:**
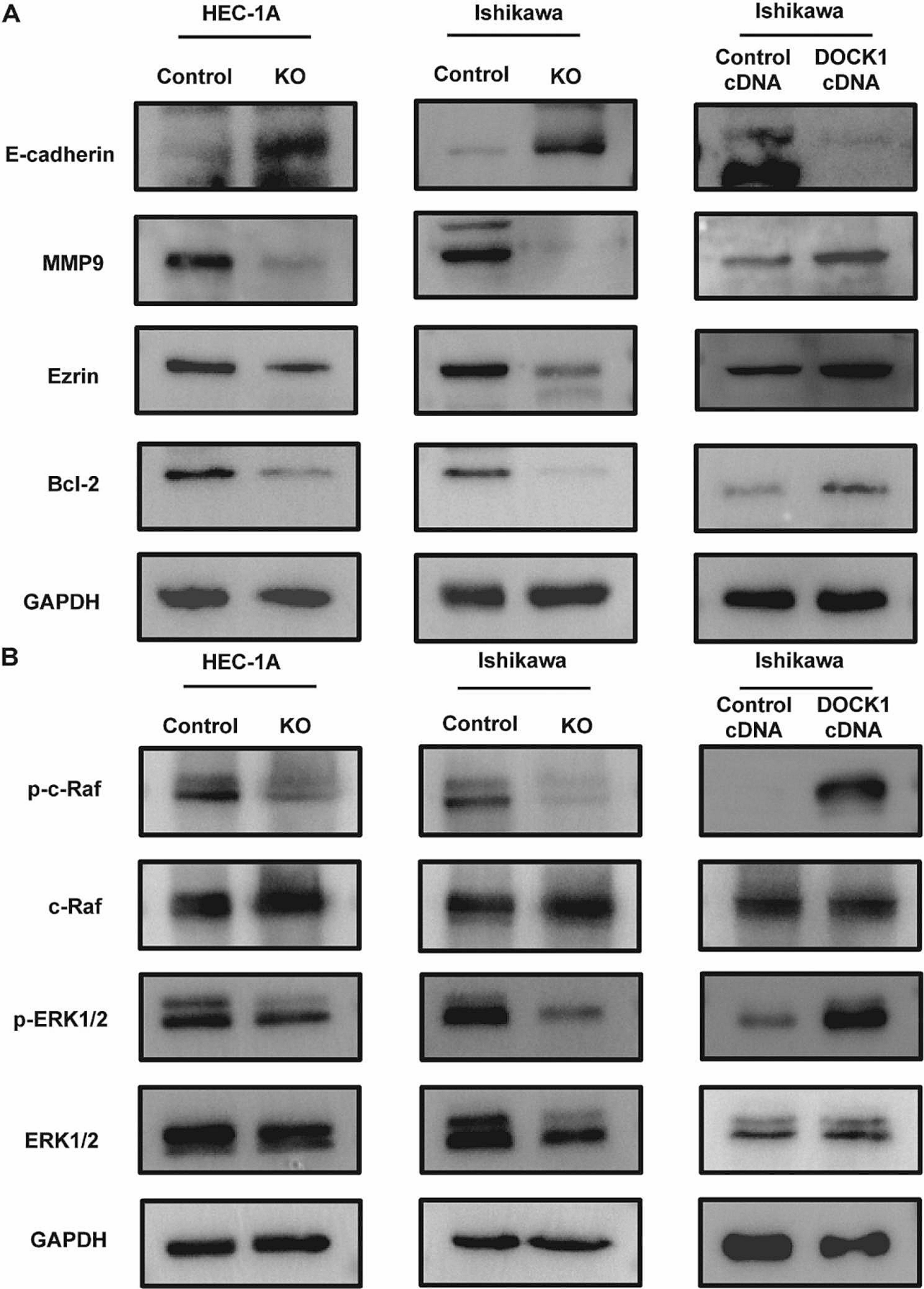
DOCK1 modulated the expression of related proteins and signaling pathway of endometrial cancer cells. (**A**) The expression of E-cadherin, MMP9, Ezrin and Bcl-2 protein was analyzed after DOCK1 knockout or overexpression. (**B**) The effect of DOCK1 knockout or overexpression on c-Raf/ERK signaling pathway related protein

First, the metastasis associated proteins MMP9 and Ezrin expression levels and apoptosis-related protein Bcl-2 expression level in HEC-1 A and Ishikawa cells were decreased after DOCK1 knockout, while DOCK1 overexpression played the opposite effect (*P* < 0.05, Fig. 5A). The E-cadherin expression was upregulated after DOCK1 knockout, while overexpression of DOCK1 reversed the effect (*P* < 0.05, Fig. 5A).

**Fig. 6:**
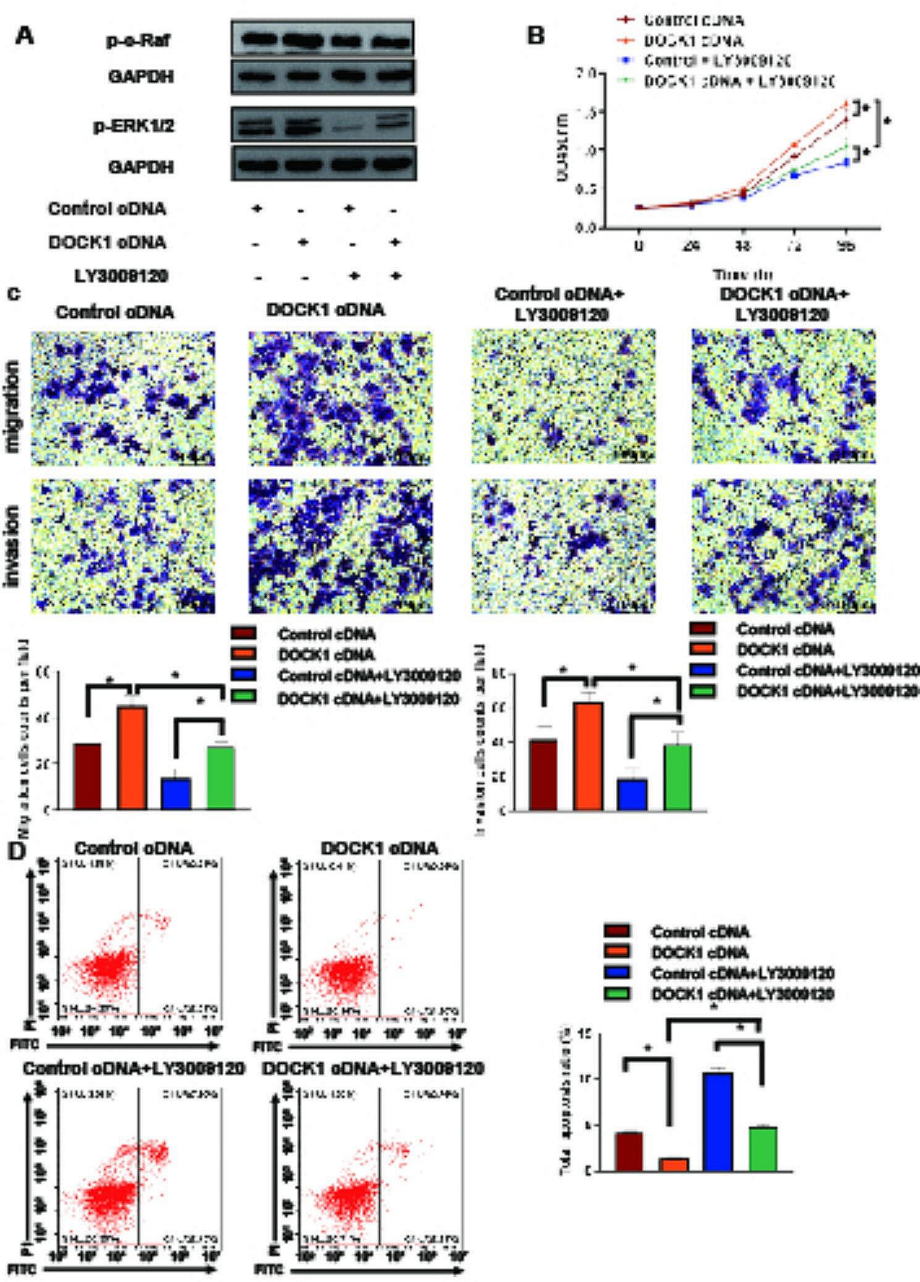
DOCK1 regulated cell viability, migration, invasion and apoptosis via c-RAF/ERK signaling pathway. (**A**) The efficacy of Raf inhibitor LY3009120 on Ishikawa cells. (**B**) The effect of LY3009120 on the viability of DOCK1-overpressing Ishikawa cells. (**C**) The effect of LY3009120 on the cell migration and invasion of cells after DOCK1 overexpression. (**D**) The effect of LY3009120 on the apoptosis of DOCK1-overexpressing Ishikawa cells. *P < 0.05

To explore the potential mechanism of DOCK1 on endometrial cancer carcinogenesis, the study further focused on whether DOCK1 mediated c-Raf/ERK pathway. The expressions of p-c-Raf and p-ERK1/2 in DOCK1 knockout groups were repressed in contrast to those in corresponding control groups, but were increased after DOCK1 overexpression (*P* < 0.05, Fig. 5B). After 20 µM Raf inhibitor LY3009120 treatment for 48 h, the expressions of p-c-Raf and p-ERK1/2 were reversed in DOCK1-overexpressing group (Fig. 6A). The malignant behavior of DOCK1-overexpressing Ishikawa cells were repressed by LY3009120 (Fig. 6B-C). In addition, the treatment of LY3009120 promoted the apoptosis of DOCK1-overexpressing Ishikawa cells (Fig. 6D). Besides, the inhibition of Rac1 activity weakened the expression of p-c-Raf and p-ERK in HEC-1 A and Ishikawa cells (Supplementary Fig. 1). The results suggested that DOCK1 might mediate c-Raf/ERK pathway via regulating the activity of Rac1 to involve in regulating endometrial cancer malignancy.

### DOCK1 knockout inhibited the growth of endometrial cancer in vivo

**Fig. 7:**
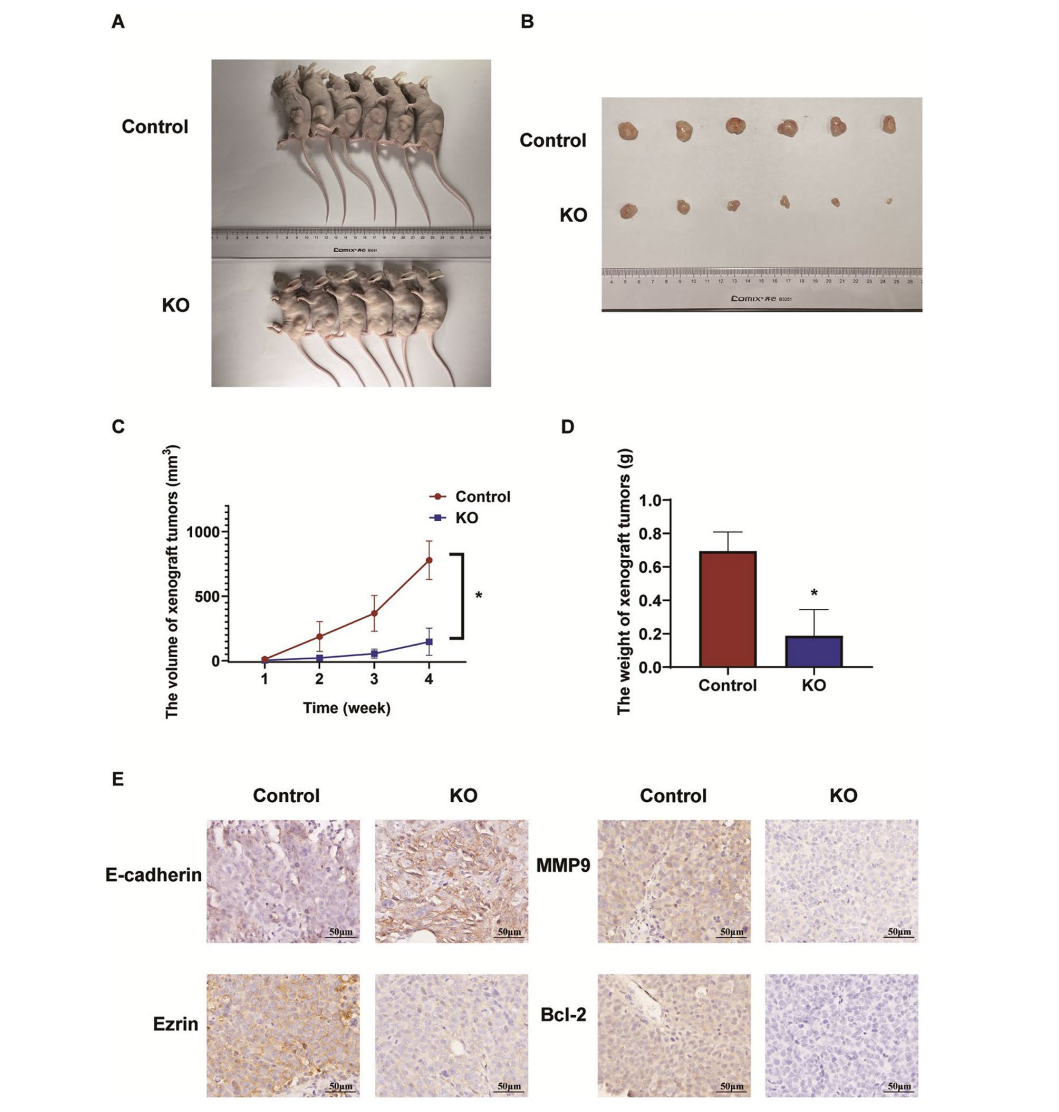
The inhibition of DOCK1 attenuated the growth of xenograft tumors in vivo. (**A-B**) The images of nude mice and subcutaneous tumors at 4 weeks after injecting DOCK1-KO Ishikawa cells. (**C-D**) The growth and weights of tumors were analyzed in DOCK1-KO group and control group. (**E**) The expression of E-cadherin, MMP9, Ezrin and Bcl-2 protein on xenograft tumors by immunohistochemical assay. *P < 0.05

To further explore the effect of DOCK1 on the progression of endometrial cancer, DOCK1 knockout and control Ishikawa cells were injected into nude mice for constructing xenograft tumor models, respectively (Fig. 7A-B). In contrast to those in control group, the volumes and weights of xenograft tumors were reduced in DOCK1 knockout group (Fig. 7C-D). Besides, E-cadherin expression was elevated, and MMP9, Ezrin and Bcl-2 expressions were decreased in DOCK1 knockout group in contrast to those in control group (Fig. 7E). Consequently, the inhibition of DOCK1 weakened the growth and induced the apoptosis of endometrial carcinoma in vivo.

## Discussion

The occurrence of endometrial cancer results from various factors, among which the activation of oncogenes promotes the development of endometrial cancer. DOCK1, a guanine nucleotide exchange factor, is dysregulated in various cancers. It was reported that DOCK1 expression was upregulated in ovarian cancer cells in contrast to that in normal ovarian epithelial cells [17]. In this work, we discovered that the expressions of DOCK1 in endometrial carcinoma tissues and cells were higher than that in adjacent tissues and normal endometrial cells.

Accumulating studies demonstrated that DOCK1 might contribute to the progression of cancer biological behavior [18, 19]. Metastasis, a significant feature of malignant tumors, often leads to poor prognosis in cancer patients [20]. It was found that the complex of DOCK1 and ELMO promoted Rac activation at the leading edge of cells, helped cells develop lamellipodia, and mediated CRK/CRKL-regulated proliferation and migration of epithelial and endothelial cells on type IV collagen. In addition, DOCK1 could modulate the phosphorylation of p130Cas and the formation of the p130Cas-Crk complex, which are key steps in regulating cell adhesion, migration and invasion [21]. Increasing studies found that DOCK1 participated in the metastasis of cancer cells. Chiang et al. [19] showed that DOCK1 accelerated the metastasis of breast cancer cells via RRP1B-Claudin-1 pathway. ELMO1 could help DOCK1 to regulate the migration ability of ovarian cancer cells [14]. Platelet derived growth factor receptor α (PDGFRα) facilitated the malignant biological behavior of glioma by promoting protein kinase A-dependent serine phosphorylation of DOCK1 [22]. Those results indicated that DOCK1 might act as an oncogene involved in the metastasis of cancer cells. Matrix metalloproteinase 9 (MMP9) redounded to the basement membrane degradation, which was an important basis for the invasion of malignant tumors [23]. The Ezrin protein encoded by the EZR gene was a membrane-cytoskeleton junction protein that could interact with a variety of growth factor receptors and adhesion molecules to mediate a variety of basic cell functions and promote tumor metastasis [24, 25]. E-cadherin, a critical epithelial marker, was proven to exert inhibitory effect on cancer metastasis [26]. In this study, DOCK1 could promote the migration and invasion of endometrial cancer cells through decreasing E-cadherin expression and upregulating MMP9 and Ezrin expressions.

Multiple signaling pathways were confirmed to take part in the mechanism of DOCK1 on mediating biological behavior of cancer. C-Raf is a kind of serine-threonine kinases and participates in the cell growth [27]. ERK, extracellular regulated protein kinases, mediates the transcriptional activation of various factors and participates in many cellular biological responses [28]. Phosphorylation of ERK is involved in the regulation of malignant biological behavior of cancer [29, 30]. It was found that serine 338 of c-Raf and threonine 202 of ERK were phosphorylated to stimulate c-RAF/ERK signaling pathway in esophageal cancer. In the present study, the expressions of p-c-Raf and p-ERK were reduced in HEC-1 A and Ishikawa cells after DOCK1 knockout. Additionally, the treatment of Raf inhibitor LY3009120 partly inhibited the effect of DOCK1 on the malignant biological behavior of endometrial cancer. It was reported that DOCK1 activated Rac1 to promote the cell molity [31] and activated Rac1 promoted MEK/ERK signaling [32]. Hence, DOCK1 might mediate c-Raf/ERK signaling pathway via Rac1 activation to modulate malignancy of endometrial cancer.

### Electronic supplementary material

Below is the link to the electronic supplementary material.

Supplementary Material 1

Supplementary Material 2

Supplementary Material 3

Supplementary Material 4

Supplementary Material 5

Supplementary Material 6

## Data Availability

The datasets used and analyzed during the current study are available from the corresponding author on reasonable request.

## Abbreviations

BSA: Bovine serum albumin
DAB: Diaminobezidin
DOCK: Dedicator of cytokinesis
EGFR: Epidermal growth factor receptor
ELMO1: Engulfment and cell motility protein 1
EMT: Epithelial-mesenchymal transition
MMP9: Matrix metalloproteinase 9
PDGFRα: Platelet derived growth factor receptor α
