# Supplementary material for: DOCK1 regulates the malignant biological behavior of endometrial cancer through c-Raf/ERK pathway
Source: BMC Cancer. 2024 Mar 4;24:296. doi: 10.1186/s12885-024-12030-1 (PMC10913561; doi:10.1186/s12885-024-12030-1)

Figure 5A The expression of E-cadherin, MMP9, Ezrin and Bcl-2 protein was analyzed after DOCK1 knockout in HEC-1A cell.

Ecadherin

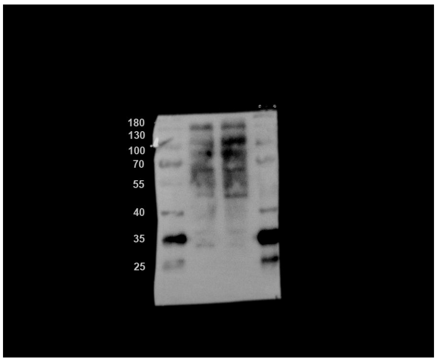

GAPDH

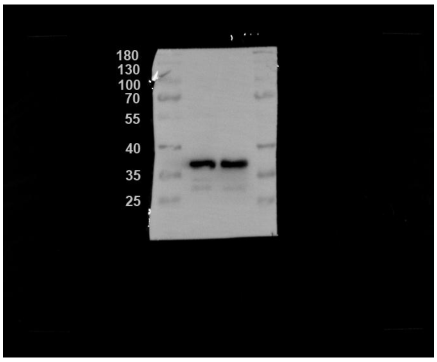

MMP9

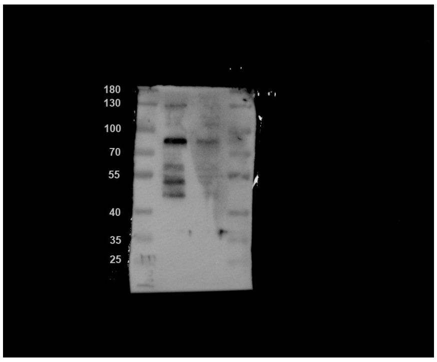

GAPDH

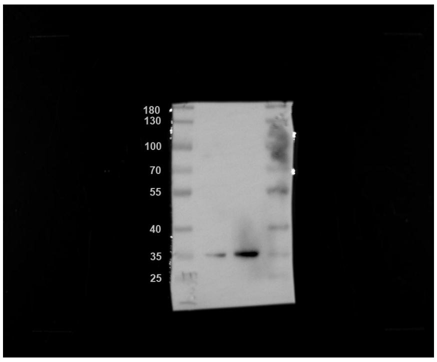

Ezrin

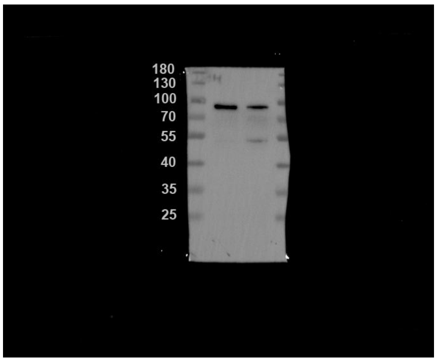

GAPDH

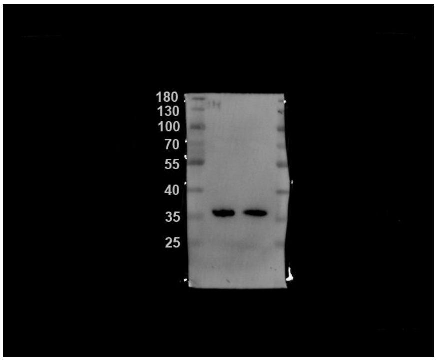

Bcl-2

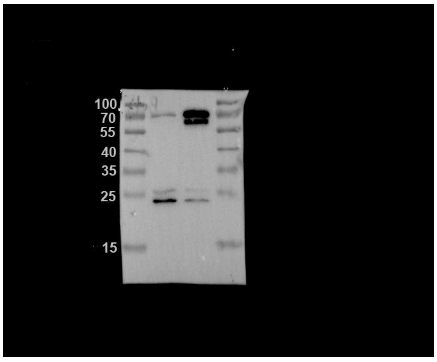

GAPDH

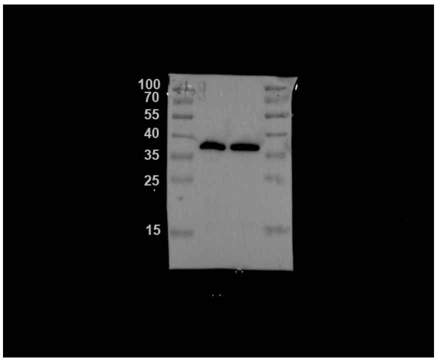

Figure 5A The expression of E-cadherin, MMP9, Ezrin and Bcl-2 protein was analyzed after DOCK1 knockout in Ishikawa cell.

Ecadherin

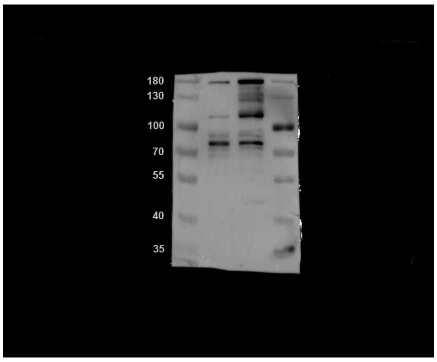

GAPDH

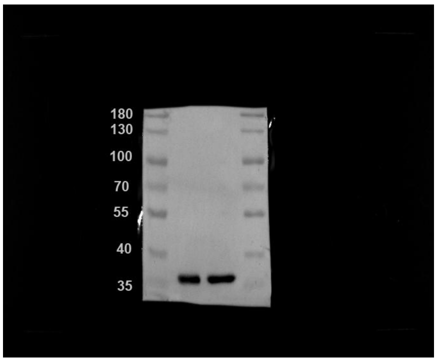

MMP9

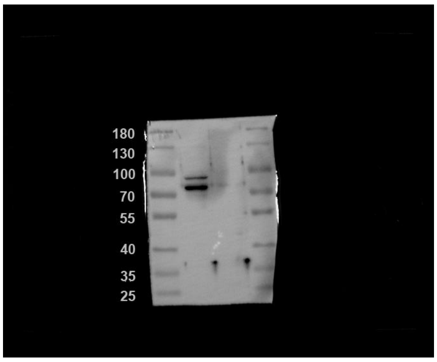

GAPDH

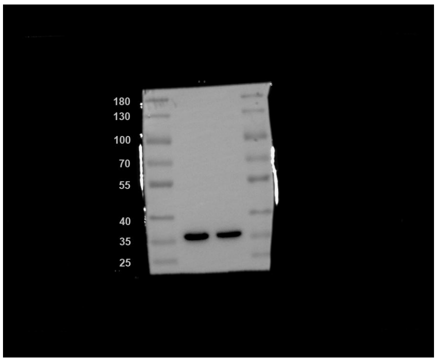

Ezrin

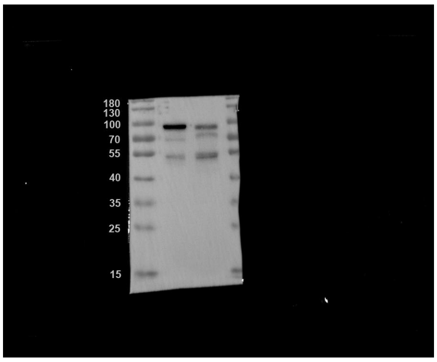

GAPDH

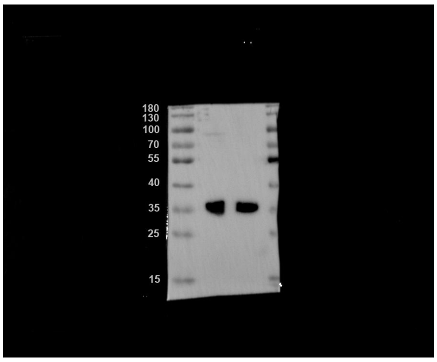

Bcl-2

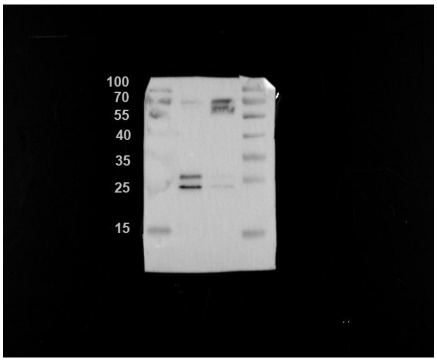

GAPDH

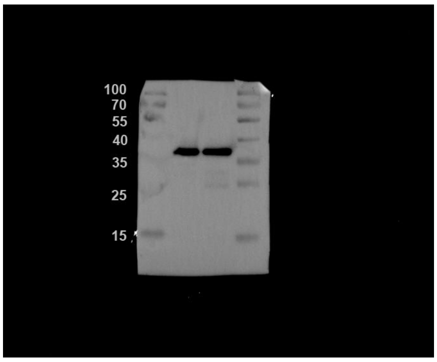

Figure 5A The expression of E-cadherin, MMP9, Ezrin and Bcl-2 protein was analyzed after DOCK1 overexpression in Ishikawa cell.

Ecadherin

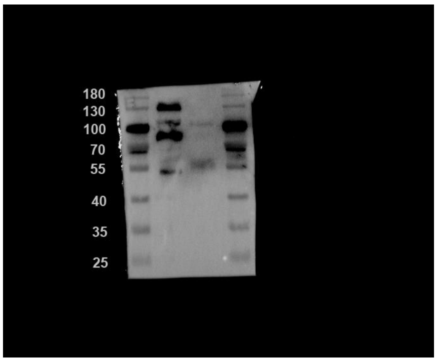

GAPDH

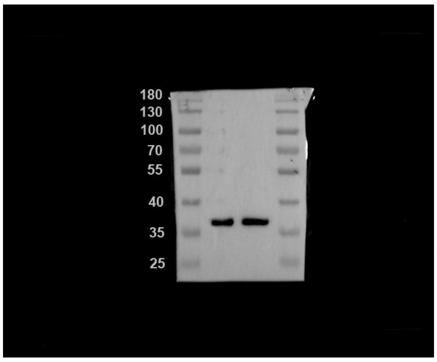

MMP9

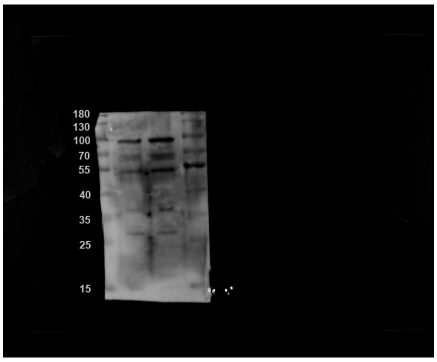

GAPDH

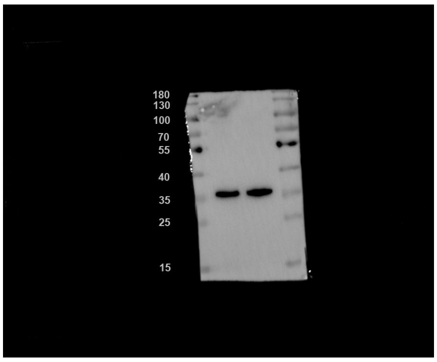

Ezrin

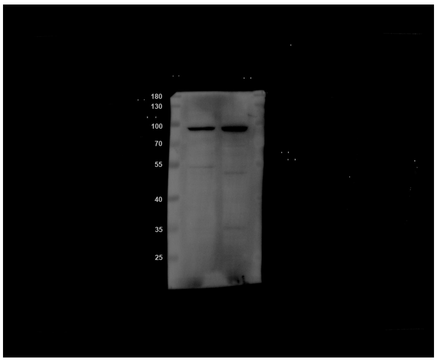

GAPDH

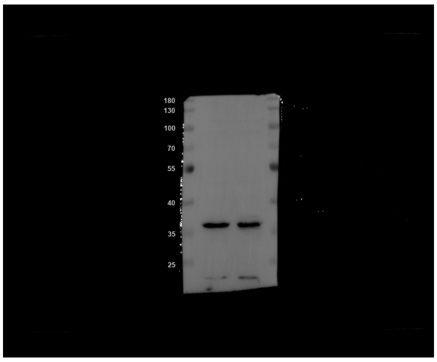

Bcl-2

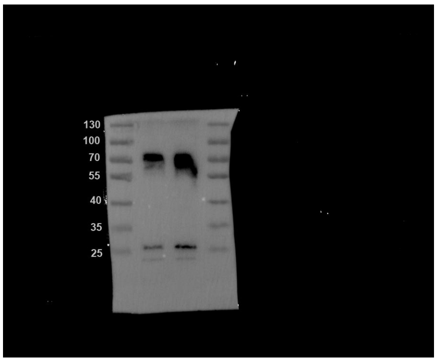

GAPDH

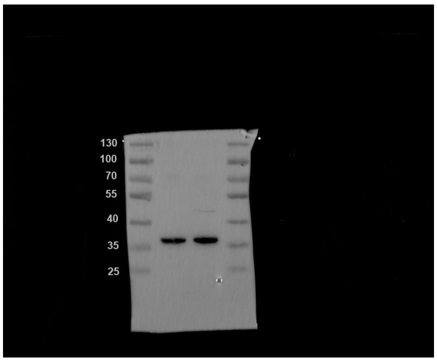

Figure 5B The effect of DOCK1 knockout on c-Raf/ERK signaling pathway related protein in HEC-1A cell.

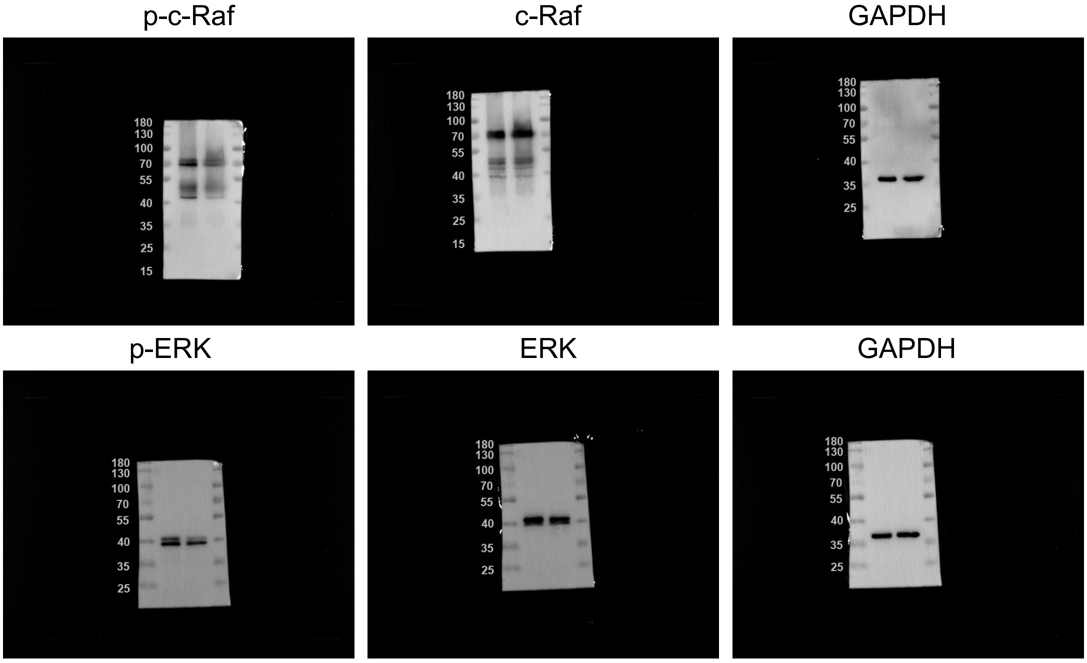

Figure 5B The effect of DOCK1 knockout on c-Raf/ERK signaling pathway related protein in Ishikawa cell.

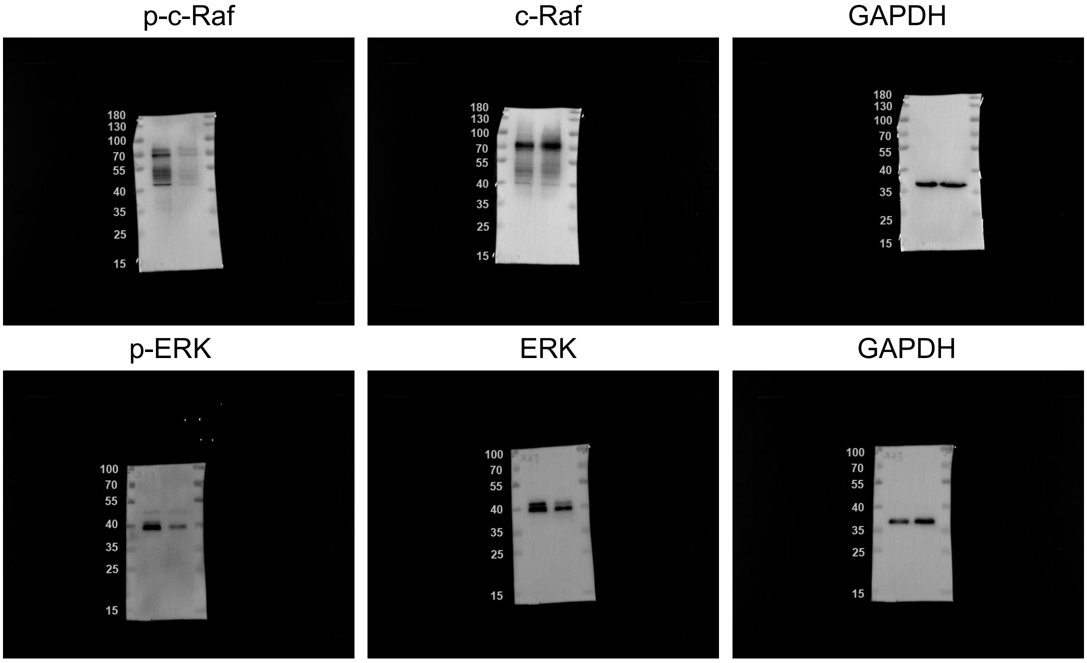

Figure 5B The effect of DOCK1 overexpression on c-Raf/ERK signaling pathway related protein in Ishikawa cell.

p-c-Raf

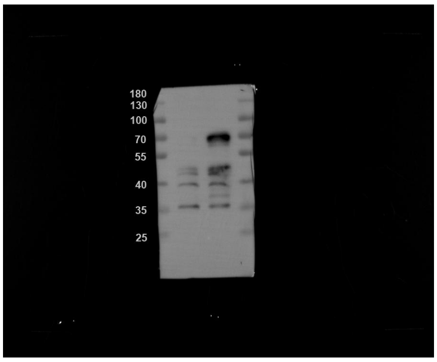

GAPDH

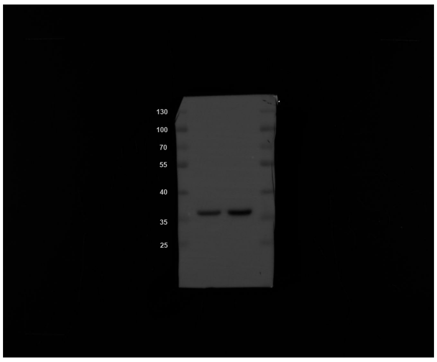

c-Raf

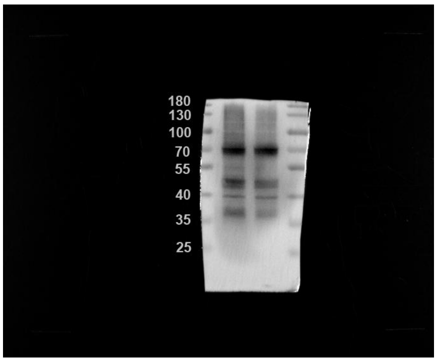

GAPDH

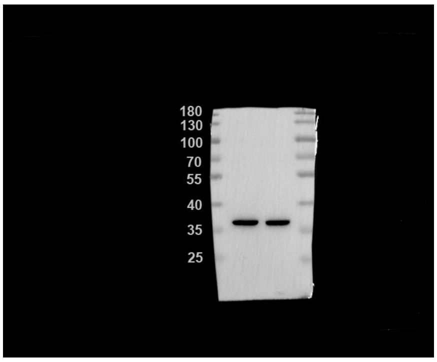

p-ERK

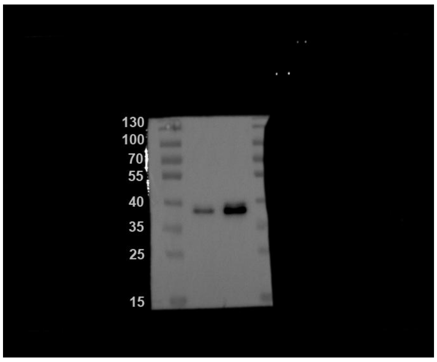

GAPDH

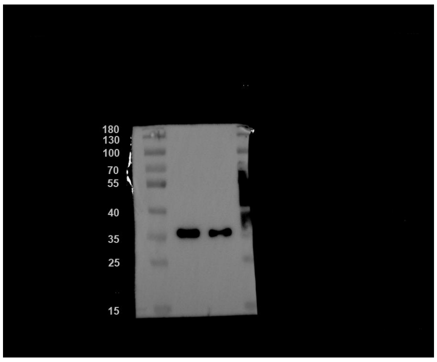

ERK

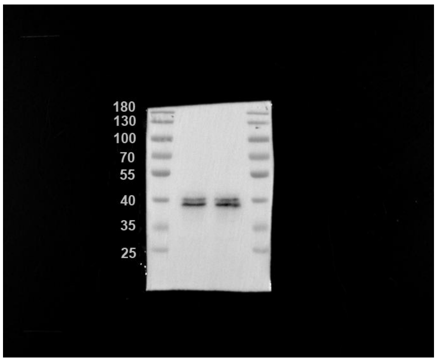

GAPDH

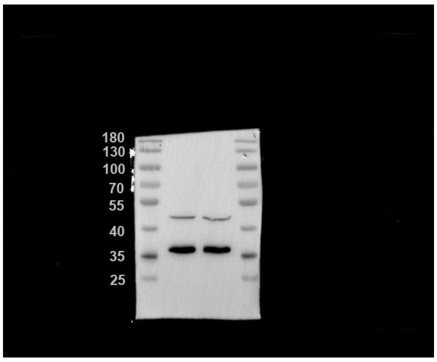

Supplement: Supplementary file 1 — Supplementary Material 1 [file 12885_2024_12030_MOESM1_ESM.pdf]
