# Supplementary figures and images for: DOCK1 regulates the malignant biological behavior of endometrial cancer through c-Raf/ERK pathway
Source: BMC Cancer. 2024 Mar 4;24:296. doi: 10.1186/s12885-024-12030-1 (PMC10913561; doi:10.1186/s12885-024-12030-1)

Figure 6A The efficacy of Raf inhibitor LY3009120 on Ishikawa cells.

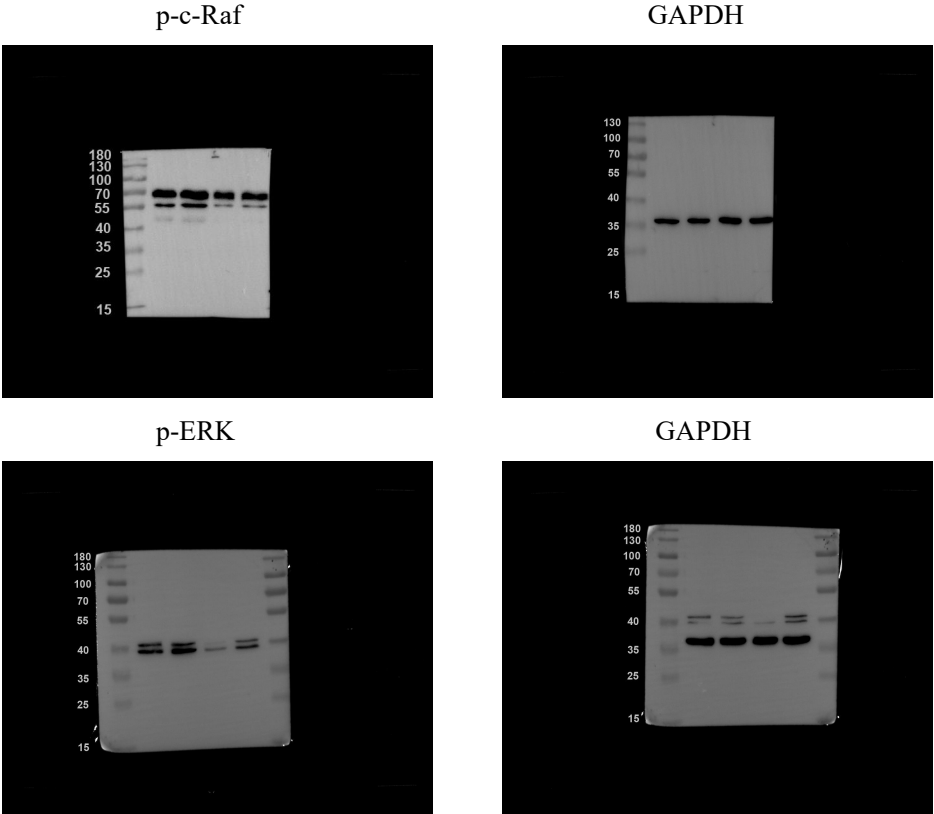

Supplement: Supplementary file 2 — Supplementary Material 2 [file 12885_2024_12030_MOESM2_ESM.pdf]
