# Supplementary material for: DOCK1 regulates the malignant biological behavior of endometrial cancer through c-Raf/ERK pathway
Source: BMC Cancer. 2024 Mar 4;24:296. doi: 10.1186/s12885-024-12030-1 (PMC10913561; doi:10.1186/s12885-024-12030-1)

Supplementary Figure 1 The efficacy of Rac1 GTPase inhibitor NSC23766 on HEC-1A cell.

p-c-Raf

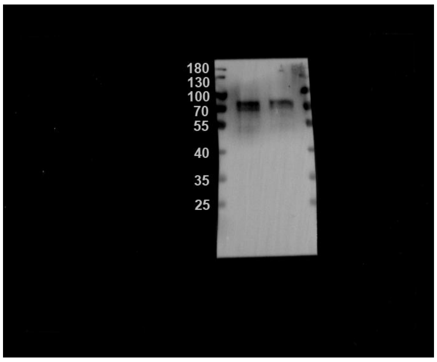

GAPDH

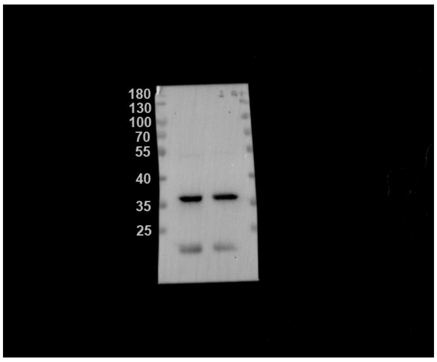

c-raf

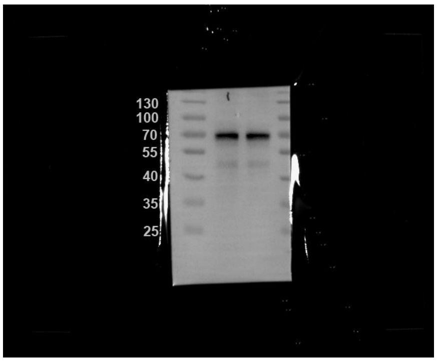

GAPDH

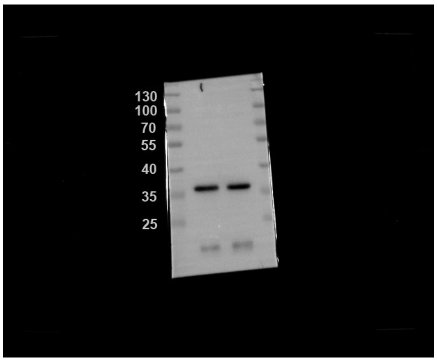

p-ERK

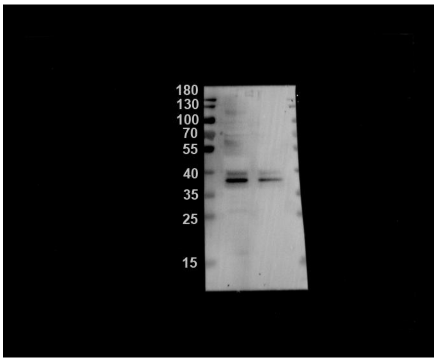

GAPDH

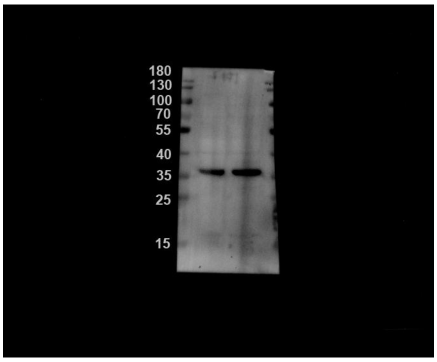

ERK

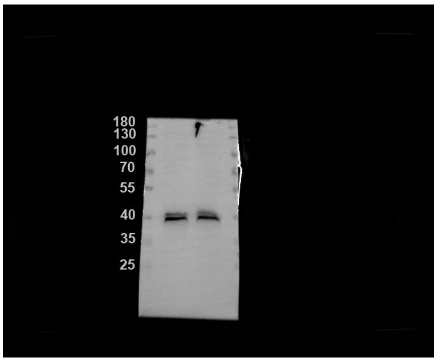

GAPDH

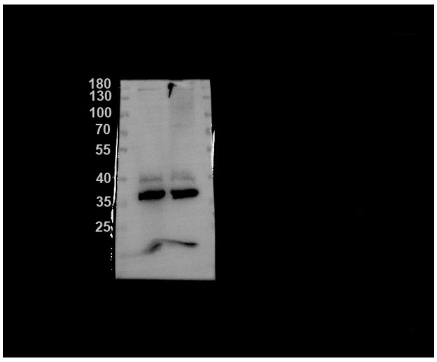

Supplementary Figure 1 The efficacy of Rac1 GTPase inhibitor NSC23766 on Ishikawa cell.

p-c-Raf

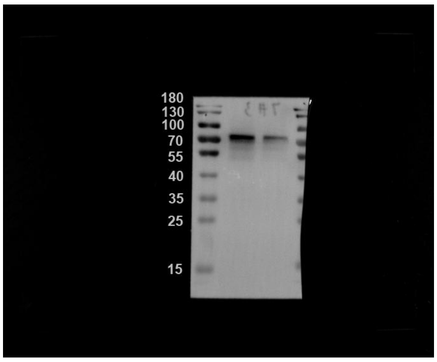

GAPDH

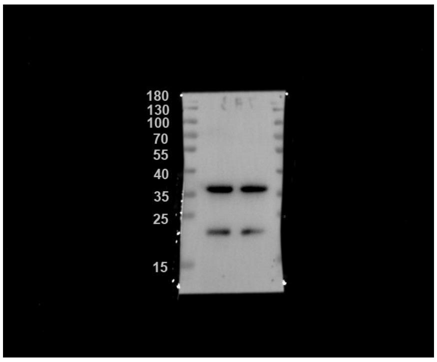

c-Raf

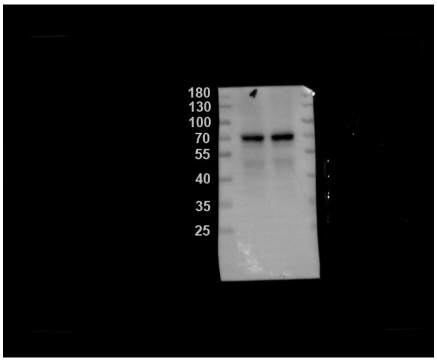

GAPDH

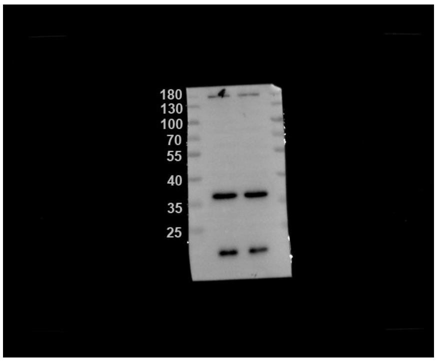

p-ERK

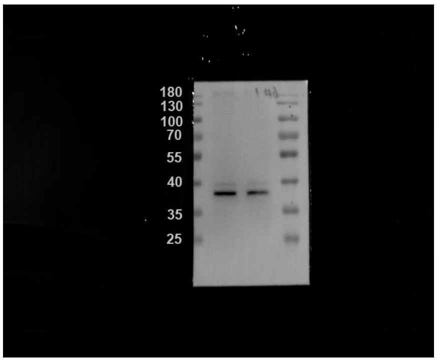

GAPDH

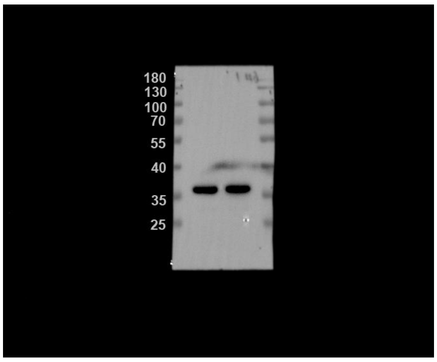

ERK

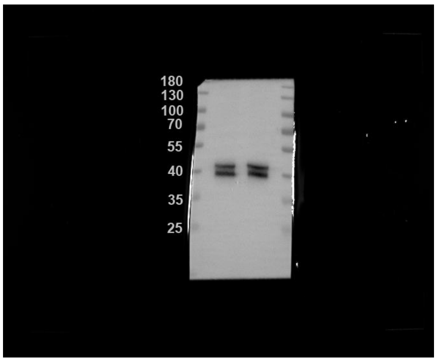

GAPDH

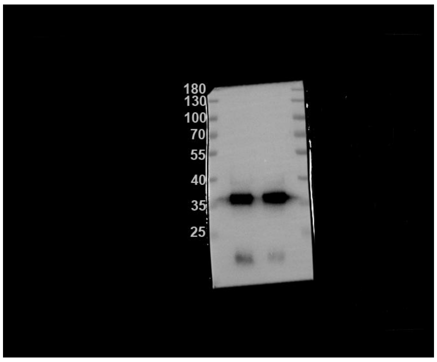

Supplement: Supplementary file 3 — Supplementary Material 3 [file 12885_2024_12030_MOESM3_ESM.pdf]
