# Supplementary material for: DOCK1 regulates the malignant biological behavior of endometrial cancer through c-Raf/ERK pathway
Source: BMC Cancer. 2024 Mar 4;24:296. doi: 10.1186/s12885-024-12030-1 (PMC10913561; doi:10.1186/s12885-024-12030-1)

Figure 1B The protein expressions of DOCK1 in EES, HEC-1A and Ishikawa cells by western blot

DOCK1 β-Tubulin

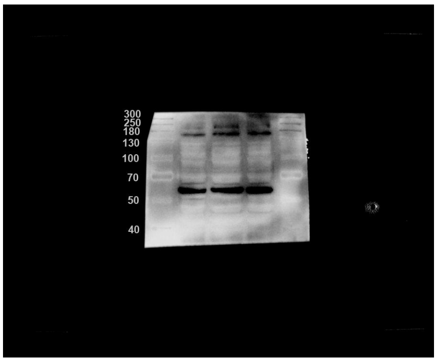

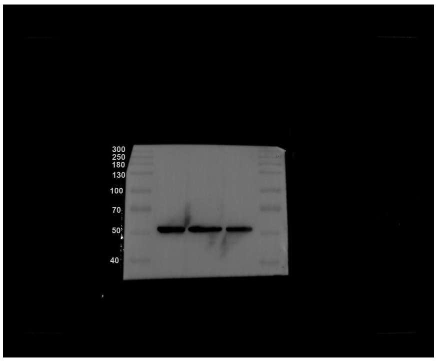

Figure 1C The efficacy of DOCK1 knockout in HEC-1A cell

DOCK1 β-Tubulin

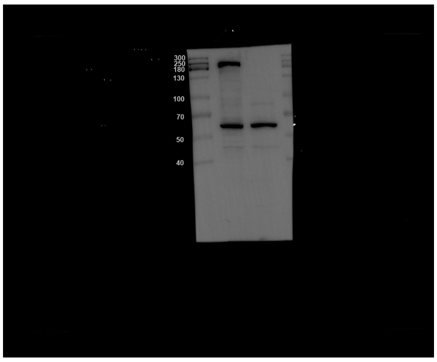

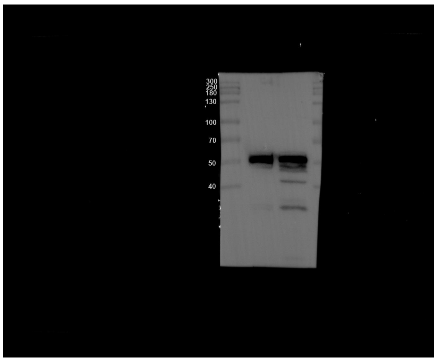

Figure 1C The efficacy of DOCK1 knockout in Ishikawa cell

DOCK1 β-Tubulin

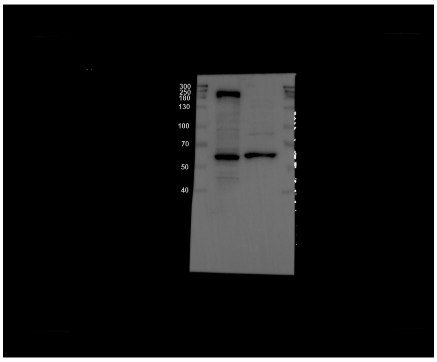

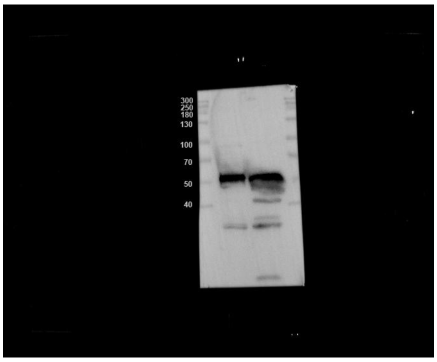

Figure 1C The efficacy of DOCK1 overexpression in Ishikawa cell

DOCK1 β-Tubulin

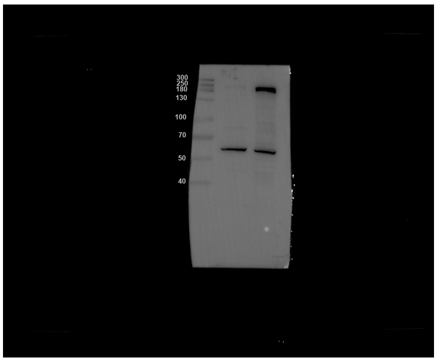

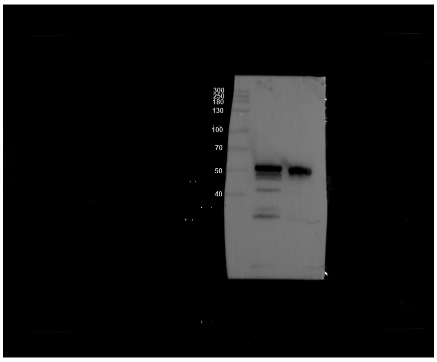

Supplement: Supplementary file 4 — Supplementary Material 4 [file 12885_2024_12030_MOESM4_ESM.pdf]
