# Supplementary material for: DOCK1 regulates the malignant biological behavior of endometrial cancer through c-Raf/ERK pathway
Source: BMC Cancer. 2024 Mar 4;24:296. doi: 10.1186/s12885-024-12030-1 (PMC10913561; doi:10.1186/s12885-024-12030-1)

Supplementary Figure 1 The efficacy of Rac1 GTPase inhibitor NSC23766 on HEC-1A and Ishikawa cells. (A) The efficacy of Rac1 GTPase inhibitor on the expression of p-c-Raf and c-Raf of HEC-1A and Ishikawa cells. (B) The efficacy of Rac1 GTPase inhibitor on the expression of p-ERK and ERK of HEC-1A and Ishikawa cells. * *P* < 0.05, ** *P* < 0.01.

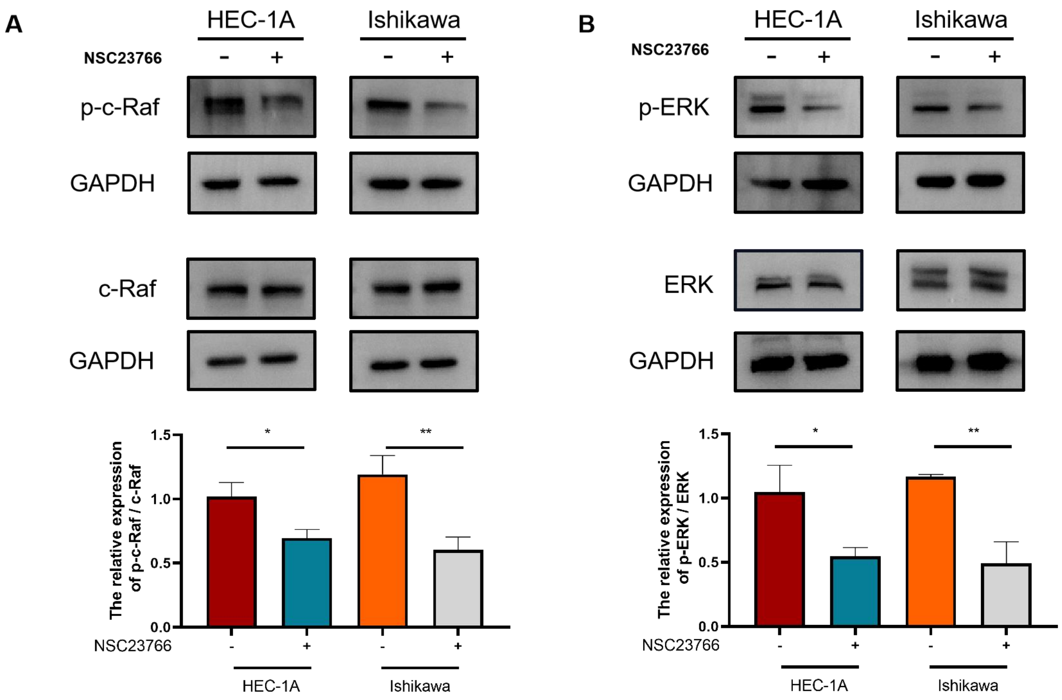

Supplement: Supplementary file 5 — Supplementary Material 5 [file 12885_2024_12030_MOESM5_ESM.pdf]
