# Supplementary material for: DOCK1 regulates the malignant biological behavior of endometrial cancer through c-Raf/ERK pathway
Source: BMC Cancer. 2024 Mar 4;24:296. doi: 10.1186/s12885-024-12030-1 (PMC10913561; doi:10.1186/s12885-024-12030-1)
Supplement: Supplementary file 6 — Supplementary Material 6 [file 12885_2024_12030_MOESM6_ESM.doc]

**Supplementary Information**

The online version contains supplementary material available at.

Additional file 1. Blots were cut prior to hybridization with antibodies.

**Figure 1C** The blots of DOCK1 and Tubulin from HEC-1A KO group, Ishikawa KO group and Ishikawa overexpressing group were from different parts of the same gel, respectively. Due to the strong effect of DOCK1 overexpression, the DOCK1 band of Control cDNA group existed but the strength was still very weak. The identity of the strong lower molecular weight band in DOCK1 immunoblots was likely to be a non-specific binding.

**Figure 5A** In HEC-1A group, the blots of Ezrin and GAPDH were from different parts of the same gel, the blots of E-cadherin, MMP9 and Bcl-2 were from different parts of the different gels and the corresponding blots of GAPDH were supplied in supplementary figure. In Ishikawa KO group, the blots of Ecadherin and GAPDH were from different parts of the same gel, the blots of MMP9, Ezrin and Bcl-2 were from different parts of the different gels and the corresponding blots of GAPDH were supplied in supplementary figure. In Ishikawa overexpressing group, the blots of Ezrin and GAPDH were from different parts of the same gel, the blots of E-cadherin, MMP9 were from different parts of the same gel and the corresponding blot of GAPDH was supplied in supplementary figure. The blots of Bcl-2 and GAPDH were from different parts of the different gels and the corresponding blot of GAPDH was supplied in supplementary figure.

**Figure 5B** In HEC-1A KO group, the blots of p-ERK, ERK and GAPDH were from different parts of the same gel, the blots of p-c-Raf and c-Raf were from different parts of the same gel and the corresponding blot of GAPDH was supplied in supplementary figure. In Ishikawa KO group, the blots of p-c-Raf, c-Raf and GAPDH were from different parts of the same gel, the blots of p-ERK and ERK were from different parts of the same gel and the corresponding blot of GAPDH was supplied in supplementary figure. In Ishikawa overexpressing group, the blots of p-ERK and GAPDH were from different parts of the same gel, the blots of ERK, p-c-Raf and c-Raf were from different parts of different gels and the corresponding blots of GAPDH were supplied in supplementary figure.

**Figure 6A** The blots of p-c-Raf and corresponding GAPDH were from different parts of the same gel and the blots of p-ERK and corresponding GAPDH were from different parts of the same gel.
